# Meta-Analysis of Differentially Expressed Genes in the Substantia Nigra in Parkinson’s Disease Supports Phenotype-Specific Transcriptome Changes

**DOI:** 10.3389/fnins.2020.596105

**Published:** 2020-12-18

**Authors:** Duong My Phung, Jinwoo Lee, SangKyoon Hong, Young Eun Kim, Jeehee Yoon, Yun Joong Kim

**Affiliations:** ^1^Department of Biomedical Gerontology, Ilsong Institute of Life and Science, Hallym University, Anyang, South Korea; ^2^Department of Computer Engineering, Hallym University, Chuncheon, South Korea; ^3^Hallym Institute of Translational Genomics and Bioinformatics, Anyang, South Korea; ^4^Laboratory of Parkinson’s Disease and Neurogenetics, Department of Neurology, Hallym University, Anyang, South Korea; ^5^Department of Neurology, Yonsei University College of Medicine, Yongin, South Korea; ^6^Department of Neurology, Yongin Severance Hospital, Yonsei University Health System, Yongin, South Korea

**Keywords:** meta-analysis, differentially expressed genes, Parkinson’s disease, disease-related genes, substantia nigra

## Abstract

**Background:**

Studies regarding differentially expressed genes (DEGs) in Parkinson’s disease (PD) have focused on common upstream regulators or dysregulated pathways or ontologies; however, the relationships between DEGs and disease-related or cell type-enriched genes have not been systematically studied. Meta-analysis of DEGs (meta-DEGs) are expected to overcome the limitations, such as replication failure and small sample size of previous studies.

**Purpose:**

Meta-DEGs were performed to investigate dysregulated genes enriched with neurodegenerative disorder causative or risk genes in a phenotype-specific manner.

**Methods:**

Six microarray datasets from PD patients and controls, for which substantia nigra sample transcriptome data were available, were downloaded from the NINDS data repository. Meta-DEGs were performed using two methods, combining *p*-values and combing effect size, and common DEGs were used for secondary analyses. Gene sets of cell type-enriched or disease-related genes for PD, Alzheimer’s disease (AD), and hereditary progressive ataxia were constructed by curation of public databases and/or published literatures.

**Results:**

Our meta-analyses revealed 449 downregulated and 137 upregulated genes. Overrepresentation analyses with cell type-enriched genes were significant in neuron-enriched genes but not in astrocyte- or microglia-enriched genes. Meta-DEGs were significantly enriched in causative genes for hereditary disorders accompanying parkinsonism but not in genes associated with AD or hereditary progressive ataxia. Enrichment of PD-related genes was highly significant in downregulated DEGs but insignificant in upregulated genes.

**Conclusion:**

Downregulated meta-DEGs were associated with PD-related genes, but not with other neurodegenerative disorder genes. These results highlight disease phenotype-specific changes in dysregulated genes in PD.

## Introduction

Parkinson’s disease (PD) is the second most common neurodegenerative disease. Loss of dopamine-producing neurons in the substantia nigra (SN) and Lewy body pathology are the pathological hallmarks of PD. Clinically, PD is characterized by bradykinesia, resting tremors, rigidity, postural instability, and gait disturbance, all of which can be reversed by dopamine replacement therapy (Kalia and Lang, 2015). While most cases of PD are sporadic, 10–15% of patients with PD have a family history of the disease. Genome-wide association studies (GWAS) and meta-GWAS of idiopathic PD led to the discovery of 90 PD risk alleles across 78 genomic regions, which explain 16–36% of the heritable risk of PD (Nalls et al., 2019). For familial PD, 17 causative genes, which were assigned the prefix “PARK,” were identified. Studies on the molecular functions of these genes have provided a comprehensive picture of PD pathogenesis, focusing particularly on the degeneration of dopaminergic neurons. Mutations in these familial PD genes cause changes in molecular functions due to loss-of-function or toxic-gain-of-function, which leads to alterations in cellular systems and pathways. Altered gene expression is regarded as a secondary change rather than the direct effect of mutations. Interestingly, the downregulation of a few PARK genes was reported in microarray studies (Moran et al., 2006; Simunovic et al., 2009). Moreover, a recent study showed that not only PARK genes but also causative genes for hereditary disorders accompanying a phenotype of parkinsonism (non-PARK genes) were also dysregulated in the SN (Kim et al., 2017). Whether dysregulated genes in the SN of patients with PD are associated with disease-causing or risk genes for other neurodegenerative disorders, such as Alzheimer’s disease or hereditary progressive ataxia, has not been investigated.

Genome-wide expression studies (GWES) to detect differentially expressed genes (DEGs) in the blood or brain tissues of animal models or patients with neurodegenerative disorders have provided insights into genes, pathways, and molecular mechanisms that are involved in neurodegeneration (Hauser et al., 2005; Soreq et al., 2013; Hokama et al., 2014). However, these studies were limited by small sample sizes and poor replication of results, and meta-analyses of DEGs are expected to overcome these limitations (Borrageiro et al., 2018). Such DEG meta-analyses have confirmed that pathways involved in synaptic vesicle cycling, dopamine receptor signaling, cellular respiration, and mitochondrial dysfunction are dysregulated in PD (Soreq et al., 2012; Glaab and Schneider, 2015; Mariani et al., 2016; Wang et al., 2017; Su et al., 2018; Kelly et al., 2019). While some GWES reported shared pathways among neurodegenerative disorders (Wang et al., 2017; Kelly et al., 2019), at least one study supports the involvement of disease-specific networks (Su et al., 2018). For example, inflammation and microglial activation are common pathological features among neurodegenerative disorders (Stephenson et al., 2018); however, activation of microglia-related genes is well documented in Alzheimer’s disease GWES, but not in PD GWES. Recently, genes enriched in particular central nervous system (CNS) cell types were also identified by RNA sequencing after immunopanning (Zhang et al., 2014, 2016). Nevertheless, analysis of DEGs in PD with regard to cell type-enriched genes has not been performed. Here, we performed a meta-analysis of DEGs (meta-DEG) in the SN of patients with PD and characterized the corresponding disease-related and cell type-enriched DEGs.

## Materials and Methods

### Cohorts and Datasets

Six microarray datasets were downloaded from the NINDS data repository after a systematic search was performed in PubMed and the Gene Expression Omnibus^1^ on January 6, 2019 using the following search terms: “Parkinson’s disease AND substantia nigra”; “Parkinson’s disease AND transcriptome”; and “Parkinson’s disease AND microarray.” Only original datasets obtained using Affymetrix chip platforms and comprising samples derived from the SN tissue of healthy individuals and patients with PD were included. The information in the six datasets included in this study is summarized in Table 1 (Grunblatt et al., 2004; Hauser et al., 2005; Zhang et al., 2005; Moran et al., 2006; Papapetropoulos et al., 2006; Duke et al., 2007; Moran and Graeber, 2008; Zheng et al., 2010; Durrenberger et al., 2012).

**TABLE 1:** Summary of the microarray datasets used in this study.

PMID	Datasets	Platform	Total no. of samples	No. of cases	
				Control	Disease
15455214	GSE20333	HG-FOCUS	12	6	6
17193926	GSE7621	HG-U133_Plus_2	25	9	16
16344956	GSE8397	HG_U133A	39	15	24
15956162	GSE20164	HG_U133A	11	5	6
15965975; 20926834	GSE20292	HG_U133A	29	18	11
20926834	GSE20163	HG_U133A	17	9	8

*PMID, PubMed ID; No, Number.*

### Meta-DEG

**FIGURE 1:**
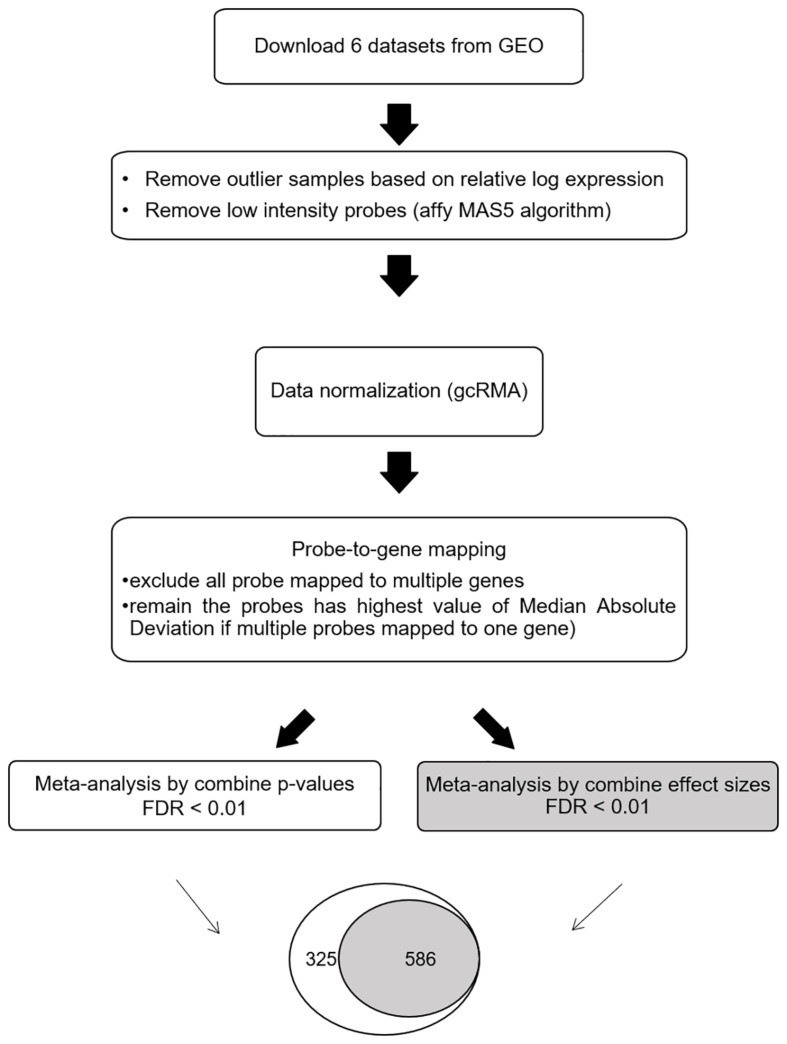
Workflow for the meta-analysis of differentially expressed Parkinson’s disease genes. Abbreviations: GEO, gene expression omnibus; FDR, false discovery rate.

The meta-DEG workflow is summarized in Figure 1. Based on the relative log expression plot, the outlier samples were detected and removed using the R package (version 3.6) arrayQualityMetrics v.3.44.0 (Kauffmann et al., 2009; Kauffmann and Huber, 2010) (Supplementary Figure 1). The Affymetrix Microarray Suite 5 algorithm was used to remove low-intensity probes in each dataset that were identified as absent. Briefly, downloaded data were normalized using the R package gcRMA v. 2.58.0 (Supplementary Figure 2). For probe-to-gene mapping, each probe in each dataset was converted into the corresponding gene. Probes mapped onto more than one gene were removed. To combine the expression levels of multiple probes mapped onto one gene, the median absolute deviation, which is a measure of dispersion robust to outliers, was applied. After preprocessing, common genes (*n* = 3,673) across all six datasets were used for the main meta-analysis as previously (Cruz-Monteagudo et al., 2016; Feng and Wang, 2017). Meta-DEGs were performed using the R package MetaMA v. 3.1.2; this package consists of 12 functions and is designed specifically for microarray meta-analysis (Marot et al., 2009). Meta-analyses were separately performed by combining *p*-values and by combining effect size in the random effect model. For combining *p*-values, a one-tailed *t*-test was performed for each gene in each study to compute the corresponding *p*-values. After the Benjamini–Hochberg correction was used, the inverse normal method was applied to combine the *p*-values. For combining effect size, the moderated effect size and its variance were combined across multiple studies using the EScombination function. *P*-values were calculated from the combined statistics using a normal distribution and adjusted using the Benjamini–Hochberg method. Fold change was calculated as log2 ratio of the mean expression of the cases and the controls. For secondary analyses, overlapping genes obtained by using both the combining effect size and combining *p*-value methods (SN-meta-DEGs) were used.

### Gene Ontology and Pathway Analysis

To embed the SN-meta-DEGs within biological networks, NetworkAnalystTM (Xia et al., 2015) was used. All, upregulated, and downregulated SN-meta-DEGs, along with their meta log fold change expression values, were separately analyzed. Enrichment networks analyzed included Kyoto Encyclopedia of Genes and Genomes (KEGG) pathways and Gene Ontology: Biological process, Gene Ontology: Molecular function, and Gene Ontology: Cellular component domains.

### Overrepresentation Analysis

The enrichment of SN-meta-DEGs in a specific gene set of interest, such as cell type-enriched genes or disease-related genes, was tested using the hypergeometric test. Then, to examine the trend of expression of cell type-enriched genes in the SN-meta-DEGs, one-sample *t*-tests were performed from the log2 fold change as previously described (Itoh and Voskuhl, 2017). Gene sets of interest were curated as follows:

#### Cell Type-Enriched Genes

Cell type-enriched genes expressed in five types of cells (astrocytes, microglia, oligodendrocytes, neurons, and endothelial cells) in the human CNS were derived from published data (Zhang et al., 2016). Differential expression was calculated as the ratio of the fragments per kilobase of transcript per million mapped reads (FPKM) of a given cell type to the average FPKM of all other cell types. Genes with FPKM < 20 were excluded. A more than fourfold change in expression of a gene in a given cell type was considered as cell type-enriched gene expression (Zhang et al., 2014). For microglia-enriched genes, an additional gene set derived from aged human brains was used (Olah et al., 2018).

#### Mendelian Disorder Genes

Causative genes for Mendelian disorders were derived from the Online Mendelian Inheritance in Man (OMIM) (Amberger et al., 2015). Gene assigned phenotypes with a known molecular basis (i.e., the phenotype mapping key “3” and the phenotype symbol “#” for phenotype descriptions with a known molecular basis) were extracted from morbidmap.txt and mimTitiles.txt. Duplicated genes, genes on mitochondrial DNA or the Y chromosome, or genes that were susceptible to a phenotype but not causing any phenotype with a known molecular background were all excluded. As a result, 4,210 genes for 6,172 Mendelian disorders were obtained.

#### Genes Related to Neurodegenerative Disorders (PD, Parkinsonism, Alzheimer’s Disease, or Hereditary Progressive Ataxia)

For constructing these gene sets, first, a set of Mendelian genes accompanying the phenotype of parkinsonism regardless of the phenotype prefix, PARK (“hereditary parkinsonism genes”) was constructed based on manual curation of the OMIM database and the authors’ knowledge (Supplementary Table 1). In brief, genes were systematically retrieved by using the keyword “parkinson^*^” in the OMIM database and then manually curating all retrieved records by searching their text for keywords with the prefix “#.” Three genes, *DNAJC13*, *TMEM230* (which are controversial for the PARK21 loci: OMIM ID,%616361), and *LRP10*, which were recently reported in familial PD and dementia with Lewy bodies without assignment of PARK loci (Giri et al., 2017; Quadri et al., 2018), were not included in our custom gene set. Conversely, two additional genes, *ATM* and *OPA1*, which have a well-documented association with the parkinsonism phenotype in the literature but were missed in the OMIM database, were included in the hereditary parkinsonism gene set (Carelli et al., 2015; Levy and Lang, 2018). As a result, a total of 92 genes belonging to hereditary parkinsonism gene sets were curated. The second gene set of PD risk genes was retrieved from the National Human Genome Research Institute-European Bioinformatics Institute (NHGRI-EBI) catalog of published GWAS (Buniello et al., 2019); a total of 381 associated single nucleotide variants (SNVs) and 280 mapped genes were derived from 44 GWAS. Another PD risk gene set, which consists of 90 variants and 86 genes, was sourced from a meta-GWAS of 17 PD datasets (Nalls et al., 2019). As a disease control gene set to test disease-phenotype specificity, four sets of risk genes for Alzheimer’s disease were used. First, the NHGRI-EBI catalog of published Alzheimer GWAS was used (MacArthur et al., 2017), and a total of 805 genes in or near 1,101 SNVs derived from 66 GWAS were curated. Next, three meta-GWAS for Alzheimer’s disease were considered. The first meta-GWAS confirmed the associations between nine previous GWAS-defined associations and 12 new genome-wide loci, presenting a total of 23 genes (Lambert et al., 2013). The second meta-GWAS for Alzheimer’s disease identified 29 significantly associated regions presenting 33 genes (Jansen et al., 2019). The third meta-GWAS for late-onset Alzheimer’s disease presented 33 genes from 19 known risk loci and 13 new risk loci (Kunkle et al., 2019). Last, a gene set for hereditary progressive ataxia was derived from a recent study in which a manually curated Mendelian gene set for progressive ataxia (*n* = 71) was constructed based on PubMed and OMIM (Eidhof et al., 2019). Since 12 genes were common between our hereditary parkinsonism and hereditary progressive ataxia gene sets, 59 ataxia genes were used for enrichment analyses (Supplementary Table 1).

### Statistical Analysis

Statistical analysis was performed using R statistical software. For overrepresentation analysis, the hypergeometric test was used, and *p*-values < 0.05 were considered statistically significant. The Bonferroni correction was used for the correction of multiple comparisons in the overrepresentation analyses of multiple gene sets. For pathway or Gene Ontology enrichment analysis, adjusted *p*-values < 0.05 were considered significant.

## Results

### Analyses of Pathways and Gene Ontologies of the SN-Meta-DEGs

By using the meta-DEG *p*-value combining method, we found 911 DEGs across all datasets, with 637 downregulated and 274 upregulated genes. Using the meta-DEG effect size combining in the moderation effects method, we identified 586 DEGs, with 449 downregulated and 137 upregulated genes. All DEGs identified from the moderation effects method were shared with those from the *p*-value combining method; therefore, 586 common DEGs were targeted for use in the subsequent analyses (Figure 1 and Supplementary Table 2). A comparison of our SN-meta-DEGs with those of a recent meta-DEG study (Kelly et al., 2019) revealed 329 (67.6%) common genes. All the common DEGs had the same direction of fold change. Analyses of enriched pathways and gene ontologies for all, down-, or upregulated SN-meta-DEGs are summarized in Supplementary Tables 3–6. Overall, the enriched pathways and gene ontologies are similar to those in the recent meta-DEG study (Kelly et al., 2019). Enriched pathways for the up- and downregulated SN-meta-DEGs were different from each other.

### Relationships Between the SN-Meta-DEGs and Cell Type-Enriched Gene Expression

**FIGURE 2:**
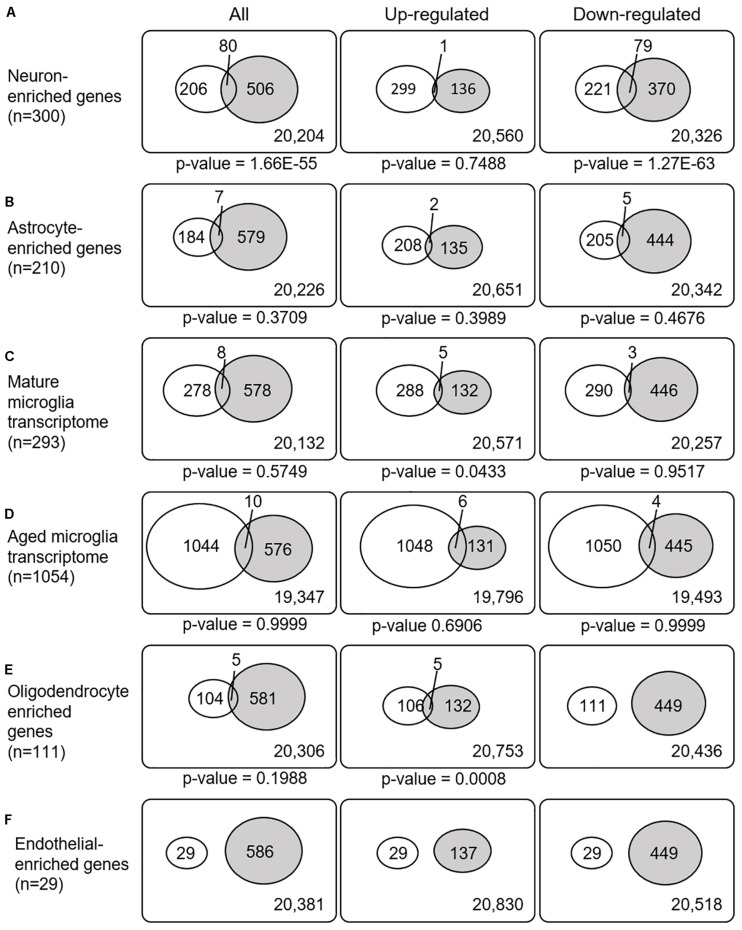
Venn diagrams illustrating the overlap between cell type-enriched genes and meta-DEGs in the substantia nigra. All *p*-values are nominal.

**FIGURE 3:**
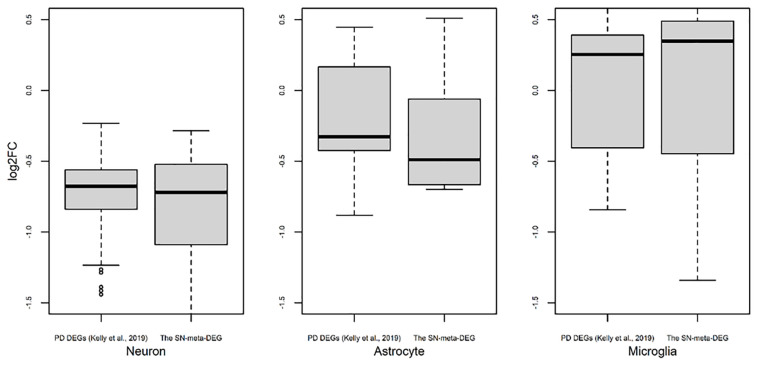
Trend of expression of cell type-enriched genes present in differentially expressed genes found in meta-analysis. Box plots for the log2 ratios of gene expression levels between disease and control samples were plotted for neurons, astrocytes, and microglia. For comparison of results of our meta-DEGs (SN-meta-DEGs), DEGs from a published study, PD DEGs (Kelly et al., 2019) were used. *P*-values for the one-sample *t*-test with these PD DEGs were 2.2E–16 for neurons, 0.0603 for astrocytes, and 0.5752 for microglia.

Recently, cell type-enriched gene expression in the CNS was reported (Zhang et al., 2016). Analyses of cell type-enriched genes using published human RNASeq transcriptome data of 21,661 genes with a cutoff at fourfold increase revealed 300 neuron-enriched genes, 210 astrocyte-enriched genes, 323 microglia-enriched genes, 111 oligodendrocyte-enriched genes, and 29 endothelial-enriched genes (Supplementary Table 7). Overrepresentation analyses of SN-meta-DEGs in cell type-enriched gene sets revealed that 477 genes (81.4%) were not enriched in a specific cell type (Table 2). Among the cell type-enriched SN-meta-DEGs (*n* = 127), 80 genes were neuron enriched, suggesting that the SN-meta-DEGs were overrepresented in terms of neuron-enriched genes (hypergeometric test, *p* = 1.59E–11) (Figure 2 and Table 2). Subgroup analysis after dividing the SN-meta-DEGs into up- and downregulated DEGs showed that statistical significance remained only in the downregulated SN-meta-DEGs. The significant downregulation of 80 neuron-enriched SN-meta-DEGs was confirmed by applying the one-sample *t*-test (*p* = 2.2E–16) (Figure 3). Comparison of the SN-meta-DEGs with the 210 astrocyte-enriched genes revealed that there were seven genes in common (hypergeometric test, *p* = 0.3709) (Figure 2 and Table 2). Further comparisons between the SN-meta-DEGs and other cell type-enriched genes, such as microglia-, oligodendroglia-, and endothelial cell-enriched genes, showed no overrepresentation of the SN-meta-DEGs in these cell types (Figure 2). To confirm the lack of association between SN-meta-DEGs and microglia-enriched genes, we compared the SN-meta-DEGs with another set of microglia-enriched genes, the human aged brain microglial-enriched genes set (*N* = 1,054), which was derived from gene expression profile RNA-seq data of microglia isolated from aged brain samples (Olah et al., 2018). There were 10 overlapping genes between the two gene sets, suggesting that the SN-meta-DEGs are not enriched in microglia-enriched genes (hypergeometric test, *p* = 0.99) (Figure 2 and Table 2). Although the mean level of gene expression of microglia-enriched genes in the SN-meta-DEGs was increased, no significant differential expression was observed when the one-sample *t*-test was applied (*p* = 0.96) (Figure 3). Re-analysis using published meta-DEG data (Kelly et al., 2019) confirmed the lack of statistical significance in terms of microglia-enriched genes (Figure 3). In the subgroup analyses, after the SN-meta-DEGs were divided into up- and downregulated DEGs, five genes (*CRYAB*, *MAP4K4*, *VCAN*, *RAPGEF5*, and *SMARCC1*) in the upregulated meta-DEGs overlapped with the oligodendrocyte-enriched genes (*p* = 0.0008). Overrepresentation analyses were marginally significant in the upregulated DEGs (nominal *p* = 0.0433), with genes enriched in mature microglia but not in aged microglia. After correction for multiple comparisons, the statistical significance remained in the neuron-enriched and oligodendrocyte-enriched genes.

**TABLE 2:** Relationships between meta-DEGs in the substantia nigra and brain cell type-enriched genes.

Intersection of meta-DEGs with	Upregulated DEGs	Downregulated DEGs
Human neuron-enriched genes	*RELN*	*TAC1, SYT1, INA, SCG2, STMN2, NELL2, GABBR2, SNAP25, VSNL1, RTN1, CHGB, GRIA1, NSF, UCHL1, SCN3B, CAP2, NSG1, PNMA2, DCLK1, NEFL, RGS4, TAGLN3, AMPH, SCG5, HPCAL4, GNG3, ENO2, CDK14, NSG2, RCAN2, RBFOX2, GOT1, STXBP1, GUCY1B1, BASP1, MAP2, RAB6B, OXCT1, ATP6V1A, MOAP1, PIP5K1B, MDH1, DNAJC12, KLC1, KIF3A, SNCA, NPTN, SYNJ1, VDAC3, OLFM1, CMAS, CHL1, CDO1, GSTA4, NDRG4, NDFIP1, MAPK9, PFN2, NDN, SLC25A4, SUB1, PCMT1, NDUFAB1, SUCLA2, CHN1, PTS, NDUFA5, IDS, MAPK10, KIFAP3, ATP6V1D, SLC9A6, GOT2, NME1, AKAP6, COX7A2L, SLC30A9, DYNLT3, G3BP2*
Human astrocytes-enriched genes	*IL17RB, CAPN2*	*SLC1A4, ANOS1, ALDH1A1, SCG3, LMO3*
Human mature microglia-enriched genes	*DUSP6, PELI1, TGIF1, NEDD9, SPRY2*	*NR4A2, CCNH, MPP1*
Human aged microglia-enriched genes	*H2BC5, TCF12, LPP, SNAP23, RPS6KA1, DAPP1*	*PYGL, ASAH1, FUCA1, NR4A2*
Human oligodendrocytes-enriched genes	*CRYAB, VCAN, RAPGEF5, MAP4K4, SMARCC1*	None
Human endothelial-enriched genes	None	None

*DEGs, differentially expressed genes; meta-DEGs, differentially expressed genes found in our meta-analysis.*

### Enrichment of Disease-Related Genes in Meta-DEG

**FIGURE 4:**
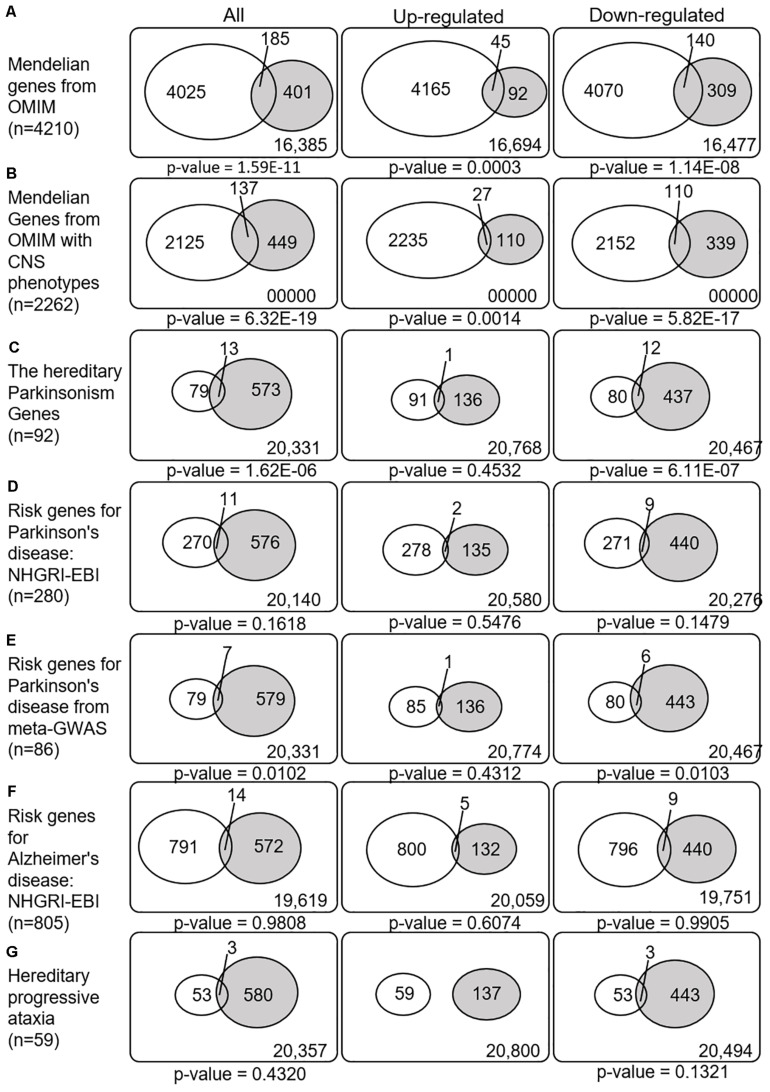
Venn diagrams illustrating the overlap between disease-related genes and meta-DEGs in the substantia nigra. All *p*-values are nominal. Abbreviations: OMIM, Online Mendelian Inheritance in Man; CNS, central nervous system; NHGRI-EBI, the Catalog of human genome-wide association studies; meta-GWAS, meta-analysis of genome-wide association studies.

We next explored whether the SN-meta-DEGs were enriched with disease-related genes. Between the SN-meta-DEGs and Mendelian disorder genes, there were 185 common genes (31.6% of the SN-meta-DEGs) (hypergeometric test, *p* = 1.59E–11) (Table 3 and Figure 4). Of these, 137 (74.1%) genes have CNS phenotypes (hypergeometric test, *p*-value = 6.32E–19) (Figure 4). Taken together, the SN-meta-DEGs were enriched with Mendelian genes, particularly those with CNS phenotypes. In the hereditary parkinsonism gene set, 12 genes were present in the SN-meta-DEG set (hypergeometric test, *p* = 1.62E–06) (Table 3 and Figure 4). Interestingly, all the overlapped genes were downregulated (Figure 4). Overrepresentation analysis with a set of PD risk genes from a meta-GWAS study (Nalls et al., 2019) was marginally significant (*p* = 0.01), but not with a set of pooled PD risk genes in the NHGRI-EBI catalog (MacArthur et al., 2017) (*p* = 0.16) (Table 3 and Figure 4). Re-analyses of the overrepresentation test of meta-GWAS PD risk genes in the down- or upregulated gene subgroups revealed that statistical significance remained only in the downregulated genes (Table 3 and Figure 4). None of four gene sets for Alzheimer’s disease was significantly enriched in the SN-Meta-DEGs: no gene in the Alzheimer’s disease meta-GWAS gene set and 14 genes in the Alzheimer’s disease NHGRI-EBI pooled risk genes overlapped with the SN-meta-DEGs (hypergeometric test, *p* = 0.98) (Table 3 and Figure 4). Last, there were three genes (*ITPR1*, *ATP8A2*, and *ATXN10*) that overlapped with a gene set of hereditary progressive ataxia (*n* = 59) and the SN-meta-DEGs (*p* = 0.4320). After correction for multiple comparisons, statistical significance of the overrepresentation analyses remained in the Mendelian genes from the OMIM, and in those with CNS phenotypes and hereditary parkinsonism genes.

**TABLE 3:** Relationships between disease-related genes and meta-DEGs in the substantia nigra of in Parkinson’s disease.

Gene sets	Up-regulated DEGs	Down-regulated DEGs
Mendelian genes from OMIM	*KCNJ2, INSR, KAT6A, PLOD3, CREBBP, CRYAB, TCF12, MYOT, RELN, PABPN1, CBS, SMC1A, DHFR, PKD1, MUTYH, ZIC1, CNOT2, ATP8B1, RELA, TAZ, RAF1, SOS2, LPP, ABL1, MEIS2, TGIF1, DUSP6, VCAN, MSX1, OPHN1, PPM1D, LBR, CDKN1C, LRP2, TGFB3, XPC, PKD2, ZBTB18, THRA, CRY1, TRPS1, TCOF1, SPRY2, SCARB1, DMPK*	*UGP2, COX7B, TACO1, IGSF1, USP9X, CRAT, SLC1A4, MAGED2, UROD, ATP1A1, PPP2R5D, VPS33B, SRP72, PCCB, SGSH, ACO2, GLRB, GYG1, CAMK2B, GNAO1, BPGM, PPP2CA, MSH2, SLC12A5, PYGL, GCLM, ATP6V1E1, GNAS, RRAS2, DNM1, SLC25A12, NNT, DNAJC12, DIABLO, SUCLA2, AFF2, OXCT1, ANOS1, GOT1, GOT2, NDUFV2, AUH, AMPD2, MRPS7, IDH3B, STAT1, SC5D, PDHX, RAP1GDS1, SLC25A4, ACAT1, F8, CSNK1D, AIMP2, NEFH, B4GAT1, UROS, OPTN, AFG3L2, NEFL, DNM1L, KATNB1, STXBP1, AP3B2, PYROXD1, SLC1A1, PEX11B, SNAP25, NDUFA10, SLC9A6, PSMB4, PDHB, NDUFA2, SMARCA4, IDS, CHN1, PLCB4, TXNL4A, TUBB4B, CACNA2D2, ATP8A2, ASAH1, GNAL, XK, PFKM, SLC30A9, GABBR2, CHCHD2, PSMD12, GFPT1, EIF2B3, ATP6V1A, FUCA1, NDN, UCHL1, TOR1A, GARS1, SPINT2, GCH1, ATXN10, ATP6V1B2, KIF2A, TBCE, SLC25A32, PLAA, PPP2R2B, NDUFS1, HARS1, TUBB3, PDXK, GSS, L1CAM, MDH2, ITPR1, OCRL, SYT1, HK1, PTS, SYNJ1, HPRT1, DLD, GALT, SNX10, CDC42, TRIM36, SNCA, TRAPPC2L, PCSK1, FIBP, NDUFA9, SCN3B, SHOC2, PRPS1, PTDSS1, RET, SLC6A3, AGTR1, GBE1, DDC, SLC18A2*
Mendelian Genes from OMIM with the CNS phenotypes	*KCNJ2, INSR, KAT6A, PLOD3, CREBBP, TCF12, CBS, SMC1A, DHFR, ZIC1, CNOT2, TAZ, RAF1, SOS2, ABL1, MEIS2, DUSP6, OPHN1, PPM1D, LBR, CDKN1C, LRP2, TGFB3, ZBTB18, THRA, TRPS1, DMPK*	*UGP2, COX7B, TACO1, IGSF1, USP9X, CRAT, SLC1A4, ATP1A1, PPP2R5D, VPS33B, PCCB, SGSH, ACO2, GLRB, CAMK2B, GNAO1, PPP2CA, MSH2, SLC12A5, ATP6V1E1, GNAS, RRAS2, DNM1, SLC25A12, NNT, DNAJC12, SUCLA2, AFF2, ANOS1, GOT2, NDUFV2, AUH, AMPD2, MRPS7, STAT1, SC5D, PDHX, SLC25A4, ACAT1, AIMP2, NEFH, B4GAT1, AFG3L2, NEFL, DNM1L, KATNB1, STXBP1, AP3B2, PYROXD1, PEX11B, SNAP25, NDUFA10, SLC9A6, PSMB4, PDHB, NDUFA2, SMARCA4, IDS, CHN1, PLCB4, TXNL4A, CACNA2D2, ATP8A2, ASAH1, GNAL, XK, SLC30A9, GABBR2, CHCHD2, PSMD12, EIF2B3, ATP6V1A, FUCA1, NDN, UCHL1, TOR1A, GCH1, ATXN10, ATP6V1B2, KIF2A, TBCE, PLAA, PPP2R2B, NDUFS1, HARS1, TUBB3, L1CAM, MDH2, ITPR1, OCRL, SYT1, HK1, PTS, SYNJ1, HPRT1, DLD, CDC42, TRIM36, SNCA, TRAPPC2L, FIBP, NDUFA9, SHOC2, PRPS1, PTDSS1, RET, SLC6A3, GBE1, DDC, SLC18A2*
The hereditary Parkinsonism genes	None	*DNAJC12, AFG3L2, CHCHD2, UCHL1, TOR1A, GCH1, PPP2R2B, PTS, SYNJ1, SNCA, SLC6A3, SLC18A2*
Risk genes for Parkinson’s disease: NHGRI-EBI	*MAP4K4, TRPS1*	*GBF1, PAM, VAMP4, CHL1, ALAS1, RIT2, GCH1, NSF, SNCA*
Risk genes for Parkinson’s disease from meta-GWAS (Nalls et al., 2019)	*MAP4K4*	*GBF1, PAM, VAMP4, RIT2, GCH1, SNCA*
Risk genes for Alzheimer’s disease: NHGRI-EBI	*RELN, ABCA8, MAP4K4, FBXL7, SCARB1*	*CHST1, PSMA1, RRAS2, MTCH2, VSNL1, STAU2, TSPAN13, NIT2, ACP2*
Risk genes for Alzheimer’s disease: meta-GWAS (Lambert et al., 2013)	None	None
Risk genes for Alzheimer’s disease: meta-GWAS (Jansen et al., 2019)	None	None
Risk genes for Alzheimer’s disease: meta-GWAS (Kunkle et al., 2019)	None	None
Hereditary progressive ataxia	None	*ITPR1, ATP8A2, ATXN10*

*Common genes between specific disease phenotype gene sets and up- or down-regulated meta-DEGs are listed. OMIM, Online Mendelian Inheritance in Man; meta-DEGs, differentially expressed genes found in our meta-analysis; meta-GWAS, meta-analysis of Genome wide association studies; NHGRI-EBI, The Catalog of human genome-wide association studies produced by collaboration between EMBL-EBI and NHGRI. References: Lambert et al. (2013); Nalls et al. (2019), Kunkle et al. (2019, and Jansen et al. (2019).*

## Discussion

By conducting a meta-analysis of six microarray datasets from SN samples of controls and patients with PD, we identified 586 meta-DEGs, consisting of 499 downregulated genes and 137 upregulated genes. The meta-DEGs were overrepresented with Mendelian genes, especially those with CNS phenotypes, and those with causative genes for hereditary disorders accompanying a phenotype of parkinsonism. Further, analyses of the cell type-enriched genes showed that downregulated genes in the meta-DEGs were overrepresented with neuron-enriched and oligodendrocytes-enriched genes. Our meta-analysis protocol is more stringent compared to previous meta-DEG studies because the common genes included in all six datasets were subjected to the meta-analyses, and we further used only the DEGs common to both methods of combining results. Despite the stringency of our method, our meta-DEG results are similar to those of a recent meta-DEG study (Kelly et al., 2019) in terms of individual genes, directions of gene expression changes of common genes, enriched pathways, and enriched gene ontologies.

Previous PD meta-DEG studies focused on analyzing perturbed pathways or gene regulatory networks to find upstream transcriptional regulators (Glaab and Schneider, 2015; Mariani et al., 2016; Wang et al., 2017; Su et al., 2018). However, none of these studies systematically analyzed the relationships between Mendelian genes or neurodegenerative disorder-related genes and meta-DEGs, except for one study in which overrepresentation of DEGs in the GWAS protein–protein interaction sub-network (Kelly et al., 2019). In contrast, we performed overrepresentation analysis directly between DEGs and gene sets of disease-related genes, which are either Mendelian genes or risk genes. We observed significant enrichment of DEGs with Mendelian genes for hereditary parkinsonism but not PD risk genes from the meta-GWAS. Noticeably, 31.6% of the SN-meta-DEGs were Mendelian disordered genes, and 74.1% Mendelian disordered SN-meta-DEGs show CNS phenotypes. Our observation that meta-DEGs were enriched in causative genes for hereditary parkinsonism but not in those for hereditary progressive ataxia or Alzheimer’s disease is novel. These results are in contrast with the findings of a recent study that has shown the shared pathways of meta-DEGs between PD and Alzheimer’s disease (Kelly et al., 2019). Given that 67.6% of DEGs are common between our study and the previous study (Kelly et al., 2019), this may not be simply due to difference in DEGs. In fact, shared pathways among neurodegenerative disorders do not necessarily mean that risk genes or Mendelian genes for neurodegenerative disorders are shared. Since purposes of two studies were different (pathway analysis vs. overrepresentation of disease-related genes), direct comparison is not possible. A recent study on gene expression profiling of 71 hereditary progressive ataxia genes showing differential expression in the cerebellum (Eidhof et al., 2019) strongly supports our results in terms of the disease phenotype specificity of meta-DEGs. Taken together, our findings of a group of dysregulated genes in the SN that is tightly linked with PD suggests that gene expression changes may be associated with selective vulnerability rather than ubiquitous gene alteration in neurodegenerative disorders. A few previous gene expression studies showed the presence of causative genes for familial PD genes in DEGs; however, they were not meta-analyses, and there was no statistical verification (Moran et al., 2006; Simunovic et al., 2009). The results of our previous study (Kim et al., 2017) are partly in line with those of the current study; however, there are differences in terms of their purposes and methods. In the previous study, gene set enrichment analysis was used to prove a hypothesis that not only PARK genes but also non-PARK genes are dysregulated in idiopathic PD. As control gene sets for other neurodegenerative disorders, gene sets in the KEGG database, which are not limited to Mendelian genes, were used. Here, we explored whether meta-DEGs identified stringently are related with causative or risk genes for three major neurodegenerative disorder phenotypes (i.e., parkinsonism, Alzheimer’s disease, and progressive ataxia). In this study, gene sets for neurodegenerative disorders were either curated manually using the OMIM database and published literature or were derived from the results of GWAS meta-analyses. Interestingly, among the three hereditary progressive ataxia genes that overlapped with our meta-DEG gene set, mutations in *ATXN10* were reported to present with levodopa-responsive parkinsonism, or reduction of dopamine transporter binding, as documented by 99mTc-TRODAT-1 SPECT (Schüle et al., 2017; Fabiani et al., 2019), although these findings were not included in the OMIM database. Subgroup analyses after dividing DEGs into up- and downregulated genes was only possible in the current study. Similar to that of other DEG studies in neurodegenerative disorders, we found a majority of downregulated DEGs. Moreover, overrepresentation analyses of PD-related genes showed stronger statistical significance in downregulated DEGs but not in upregulated DEGs. These findings suggest that dysregulated genes in the SN in PD might be related to a model of system failure in defense pathways against stress to maintain cellular integrity (Eidhof et al., 2019).

Only a few studies on neurodegenerative disorders have investigated cell type enrichment of a group of genes derived from DGE studies or GWAS (Itoh and Voskuhl, 2017; Mathys et al., 2019; Nalls et al., 2019). In microarray gene expression studies of brain tissue, an altered level of gene expression is generally reflected by the sum of gene expression change among different cell types. However, changes in the expression of genes that are enriched in a particular CNS cell type may suggest that pathways associated with a given cell type are involved in the pathogenesis of a disease. In one microarray study investigating cell type enrichment of DEGs in the brains of patients with Alzheimer’s disease, PD, or multiple sclerosis, microglia-enriched genes were upregulated, whereas neuron-enriched genes were downregulated in all three diseases. Although our results regarding neuron-enriched genes are consistent with this, those regarding microglia-enriched genes are conflicting; microglia-enriched genes were not overrepresented in PD meta-DEGs, and the mean level of gene expression changes in the microglia-enriched meta-DEGs were not significant. However, there are a number of differences in the methods used. First, our results are from meta-DEGs. Second, the brain regions studied are different (SN vs. frontal cortex). Third, the methods of curating cell type-enriched genes were different. We defined enriched genes as those with a fourfold change (Zhang et al., 2016), whereas the earlier study used the top 500 enriched genes in CNS cell types. Our results are also supported by re-analysis using published meta-DEG data (Kelly et al., 2019) which confirmed lack of statistical significance in terms of microglia-enriched genes. In a recent AD transcriptome study, cell type enrichment of DEGs was shown to vary depending on two different stages of the disease course (McQuade and Blurton-Jones, 2019). In the early pathological stage, 96% of DEGs were neuronal or microglia enriched, but this cell type enrichment was lost in the late stage, as most DEGs were expressed across cell types. Although the lack of microglia enrichment in our meta-DEGs may be attributable to the late stages of PD when the transcriptomes were obtained, our finding regarding microglia is supported by recent studies on PD. Risk loci from GWAS in Alzheimer’s disease are found in or near genes expressed most highly in microglia (McQuade and Blurton-Jones, 2019). In contrast, a recent study of PD meta-GWAS showed that genes near or in PD loci were enriched in neuronal cells, especially those in the SN, while no enrichment was found in microglia-enriched genes (Nalls et al., 2019). Interestingly, upregulated meta-DEGs were overrepresented with oligodendrocyte-enriched genes. The aggregation of alpha-synuclein in oligodendrocytes is a pathological hallmark of multiple system atrophy; however, studies on pathological alteration of oligodendrocytes in PD are limited. A recent single-cell transcriptome study of the human SN showed that common genetic risk loci in PD are associated with alterations in oligodendrocyte-specific gene expression (Agarwal et al., 2020). Among the five overlapped genes between the meta-DEGs and oligodendrocyte-enriched genes, upregulation of alphaB-crystallin encoded by *CRYAB* in brain tissue of PD has been reported (Braak et al., 2001). Taken together, these results suggest that neuroinflammation may play a less causal role in PD. Our study has the following limitations. First, transcriptome data of the SN in PD is available only after an autopsy, and this may cause an advanced stage selection bias. Second, gene expression changes are snapshots and may be effects rather than causes. Third, the stringency of our meta-DEG method might have reduced the number of genes associated with PD.

In conclusion, we found that downregulated SN-meta-DEGs were enriched in Mendelian genes with CNS phenotypes, particularly with PD-related genes, but not with other neurodegenerative disorders genes. Furthermore, neuron-enriched genes were overrepresented in the dysregulated genes. Our results highlight disease-phenotype specific changes of DEGs rather than common pathways underlying neurodegenerative disorders.

## Data Availability Statement

The datasets presented in this study can be found in online repositories. The names of the repository/repositories and accession number(s) can be found in the article/Supplementary Material.

## Ethics Statement

Ethical review and approval was not required for the study on human participants in accordance with the local legislation and institutional requirements. Written informed consent for participation was not required for this study in accordance with the national legislation and the institutional requirements.

## Author Contributions

YJK conceptualized the study, contributed to the methodology, acquired funding, conducted the project administration, and wrote and finalized the manuscript. DMP analyzed the data and wrote the original draft. JL, SH, and YEK analyzed the data. JY contributed to the methodology and analyzed the data. All authors contributed to the article and approved the submitted version.

## Conflict of Interest

The authors declare that the research was conducted in the absence of any commercial or financial relationships that could be construed as a potential conflict of interest.

## Footnotes

1 http://www.ncbi.nlm.nih.gov/geo
