# Supplementary figures and images for: Meta-Analysis of Differentially Expressed Genes in the Substantia Nigra in Parkinson’s Disease Supports Phenotype-Specific Transcriptome Changes
Source: Front Neurosci. 2020 Dec 18;14:596105. doi: 10.3389/fnins.2020.596105 (PMC7775392; doi:10.3389/fnins.2020.596105)

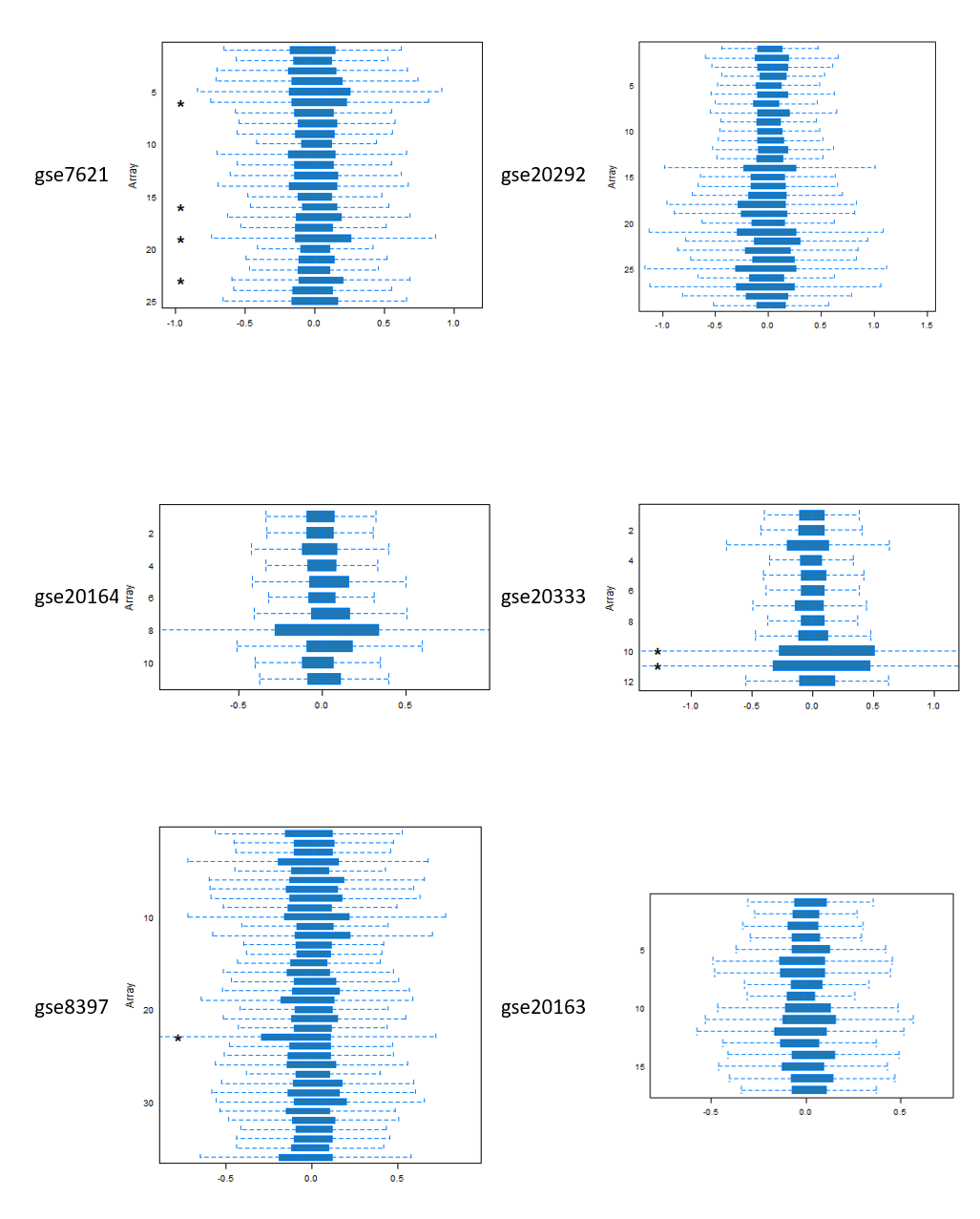

Supplement: Supplementary file 4 [file Image_1.TIFF]

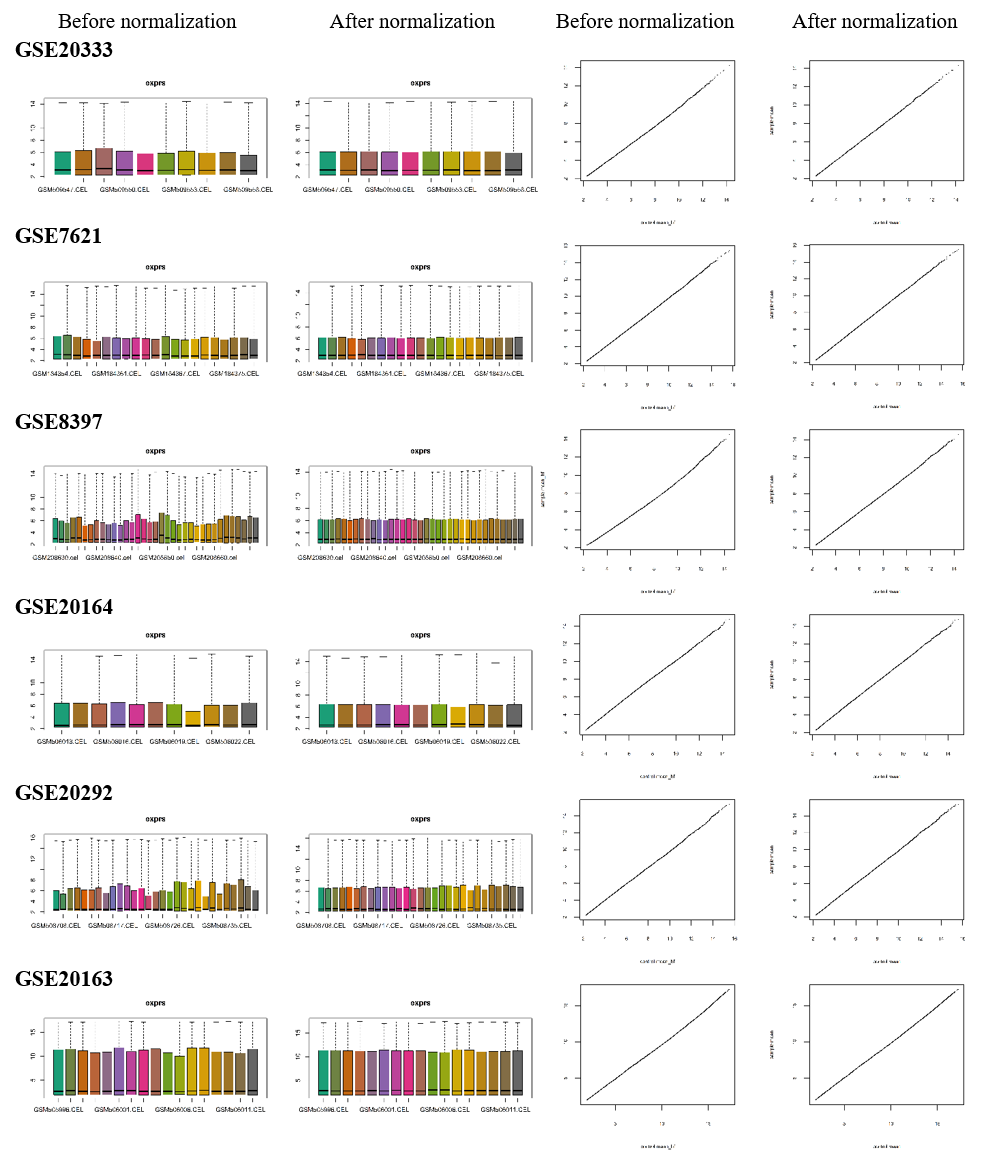

Supplement: Supplementary file 5 [file Image_2.TIFF]
